# Dental students’ concerns regarding OSPE and OSCE: a qualitative feedback for process improvement

**DOI:** 10.1038/bdjopen.2017.9

**Published:** 2017-06-09

**Authors:** Ambreen Shahzad, M Humza Bin Saeed, Sadia Paiker

**Affiliations:** 1 BDS, Islamic International Dental College, Riphah International University, Islamabad, Pakistan; 2 Department of Community Dentistry, Islamic International Dental College, Riphah International University, Islamabad, Pakistan

## Abstract

**Objectives:**

Objective structured practical examination (OSPE) and objective structured clinical examination (OSCE) have become established as reliable, valid and objective methods of assessing practical and clinical skills in dental schools. This study explored the perceptions of dental undergraduates' regarding OSPE and OSCE.

**Design and Setting:**

Two focus groups were made; the first consisted of students who had recently undergone an OSPE, while the other group was of fresh graduates (FG) who had given an OSCE in the final examination. A trained facilitator conducted the discussion-based interview for each focus group. Both discussions were recorded via audio recorders and transcribed verbatim. The data were thereafter analysed thematically.

**Results:**

Findings from the study suggested that the students and FGs were generally satisfied with the OSPE and OSCE. However, they perceived that the time allocated to the stations was not well balanced, nor were the examiners trained to conduct the examination. More importantly, the FGs opined that practical skills were not adequately tested on the OSCE, and thus the curricular content was not adequately covered.

**Conclusion:**

The study highlights issues that may arise while conducting the OSPE and OSCE, thus informing future guidelines for conducting OSPE and OSCE.

## Introduction

Assessment in medical education has undergone a revolution over the course of the past half a century. Our understanding of methods employed to assess the competencies of medical and dental students has changed dramatically as research in medical and dental education explores new horizons. These competencies have been simply explained as ‘the habitual and judicious use of communication, knowledge, technical skills, clinical reasoning, emotions, values and reflection in daily practice for the benefit of the individuals and communities being served’.^1^

Although this definition appears to be simplistic, it practically encompasses multiple dimensions which cover a diverse range of disciplines. The Accreditation Council for Graduate Medical Education (ACGME) describes six interrelated domains within medical education: (1) medical knowledge, (2) patient care, (3) professionalism, (4) communication and interpersonal skills, (5) practice based learning and environment and (6) systems-based practice.^2^ It is not only imperative, but an extremely arduous and technically sensitive task to design methods to assess domains that cover such wide areas of human competence.

The statement that ‘assessment drives learning’ is not just a saying by George E Miller that is simply quoted and unquoted to emphasise the value of assessment in medical education literature. This has taken the value of a foundation stone when it comes to designing curricula. This is because the way we test students defines not only how they study, but more importantly, what and how much they study.^3^

Assessment conveys what we deem as important and acts as a very compelling motivator of student learning.^4^ The most common division of assessment methods is based on the contribution of the assessment strategy on the overall performance score of the student. The two main categories are formative and summative.^3^ A number of methods have been used to assess clinical and practical skills, including long case exam, OSCE,^5^ mini clinical evaluation exercise,^6^ OSPE,^7^ multi-source feedback^8^ and Portfolios^9^ among others.

The traditional methods of assessing clinical and practical skills that include long case viva or the traditional oral clinical examination has its limitations. In a traditional exam students are given a single practical or clinical task to perform and their skills are tested solely on the basis of that particular task. Their communication skills and theoretical knowledge are tested in vivas that are not well structured and lack objectivity as there is no check how many questions are being asked by each student and whether they are of the same difficulty. This creates a lot of biasness and does not evaluate the students fairly. Therefore, the traditional examination has low reliability, lack of generalisability, high subjectivity, non-structuredness and biasness. Vivas are still a common and extensively used mode of assessment in our country and as described by Khan *et al.*,^10^ *viva voce* is still being widely used in different parts of the world. Ever since the middle of the twentieth century, assessment in medical and dental education has undergone a myriad of changes. As the traditional methods have been modified to cater for many of their previous shortcomings, newer methods of assessment, such as the OSCE, OSPE, multi-source feedback and mini clinical evaluation exercise have been introduced in medical and dental education.

OSCE, later extended to the OSPE, was described in 1975 and in greater detail in 1979 by Harden *et al.*^5^ Both of these are approaches for student assessment in which competencies are evaluated in a comprehensive, consistent and structured manner.^11^ OSCE/OSPE gives a summative as well as formative assessment of the students.^12,13^ The terms OSPE and OSCE are both usually applied as equivalents and with no differentiation.^14^

In an OSCE the students have to pass through a number of stations. The number of stations vary from 15–20. However, lesser number of stations has also been reported in use. Time allocated for each station varies from subject to subject but usually each station is timed from 4–5 min.^7^ One or two rest stations may also be included in certain situations.^15^ In an OSCE the student must be able to demonstrate a clinical competency, rather than merely demonstrating having theoretical knowledge of the subject. The students are then marked on each station according to a checklist or a global rating scale.^12^

AMEE (Association for Medical Education in Europe) Guide No. 81 Part I describe OSPE (as a variation of OSCE) as a method of examination used to test practical skills and knowledge in a non-clinical environment.^10^ The students have to pass through stations termed as 'response stations’. Within a limited time period the students have to respond to objective type questions, interpret data or record their findings for a given experiment. The deficiencies related to subjectivity, lack of assessment of communication skills and attitudes of students in traditional practical examination are now thought to be met well with OSPE.^7^ OSPE is now accepted as a gold standard for assessing practical lab skills worldwide.^16^ Not only does the OSPE refine students’ pre-clinical skills but it also helps to prepare them for clinical years ahead. OSPE is a valid and reliable instrument with good capacity for discriminating between different categories of students.^17^ OSCE has several advantages as follows: versatility, as it allows the stations to be tailored according to the skill that needs to be tested; objectivity, as scenarios are uniform for all candidates and marked according to a predefined checklist allowing easy recall, teaching audit and determination of standards;^18^ and broad scope, as OSCE takes a shorter time to execute, thus allowing more number of students to be assessed at a given time over a broader range of subjects.^19^ Thus, it allows for review of teaching technique and curricula.^18^ OSCE with respect to reliability, validity, objectivity and feasibility is still undergoing a continuous process of refining. However, OSCE has achieved worldwide acceptability as an established mode for learner assessment.^4,5,13^

OSPE/OSCE should be designed in such a way that it assesses the skills with validity. For instance, asking a student to write down how to give an inferior dental nerve block anaesthesia would not be a valid way of assessing the skill. Instead, the examiner should observe a student as she/he gives the anaesthesia on a patient or a manikin.^20^ Table 1 shows various skills tested in OSCE/OSPE and a valid method of assessing them.

The OSPE and OSCE may be evaluated keeping Van der Vleuten’s five-point criteria for determining the usefulness of methods of assessment. Both these methods are reliable, valid, and acceptable and have a crucial impact on the educational process. The OSPE has found to be cost-effective as well. However, the OSCE has the disadvantage of being expensive and sophisticated method of assessment. Although the reliability and validity of these two methods of assessment are evidenced by literature, there is room for great improvement since there is not a single method of assessment that has the ability to test all the required competencies thoroughly.

When the OSPE and OSCE are evaluated in more detail, several other shortcomings may be highlighted. Although the checklists and global scales are generally standardised and robust, variation in marking the candidates’ performance may be recorded due to assessor’s bias. Also, a reduction in the use of real patients’ and an increase in standardised patients in undergraduate medical OSCEs have been reported. This has resulted in setting of easily standardised stations, ‘ignoring’ less likely clinical situations. Consequently, students tend to study for such cases which are easy to standardise in the OSCE, resulted in an unintended side effect of the OSCE. Furthermore, the high reliability of the OSCE has been reported to be often achieved at the expense of their validity. Another drawback of the OSCE is that despite incompetency in a particular skill, the student can still pass the exam due to his/her performance at other stations.^3^

The limitations and shortcomings of the OSCE and OSPE warrant for strategies promoting continuous evaluation and improvement in these assessment methods. Running audit cycles or evaluation and monitoring programs are important in this regard. Student perceptions can have a pivotal role in providing information regarding improving teaching and assessment methodology.^21,22^ Students’ perceptions may be recorded through structured questionnaires or by conducting in depth interviews or focus group discussions.

This study was designed to gain insight into the students’ perceptions regarding OSPE and OSCE conducted in an undergraduate dental school in Islamabad.

## Materials and methods

The study was conducted at the dental college (IIDC) of Riphah International University (RIU), Islamabad. Ethical approval was obtained from RIU’s Institution Review Committee. (IIDC/IRC/201505001).

This qualitative study focuses on students’ reflection upon two different types of assessment methods: OSPE and OSCE. The methodology is based upon Schon’s reflection types; ‘reflection on action’.^23^ The students’ reflected upon their experiences in OSPE and OSCE in two focus group discussions.

### Participants

Students undergo an OSCE examination in their final/fourth year of BDS. Therefore, participants for OSCE focus group were recruited from FGs. This group had a total of eight; six female and two male participants. The FGs were asked about their perceptions regarding OSCE for the subjects of Orthodontics (OR) and Oral Surgery. These two particular subjects were chosen to be evaluated for this study as only these two departments were using OSCE as a mode of assessment in our institution at that time.

For the OSPE focus group, participants were selected randomly from third year BDS. A total of eight, five female and three male, participants were in this group. These students had recently given their OSPE for the subjects of Oral Biology (OB) and Community Dentistry (CD) during their second year BDS examination. Again, these two particular subjects were the only pre-clinical dental departments conducting an OSPE as a mode of assessment at our institution.

The sample size was set to optimum as described by Gill *et al.*^24^ A sample size less than that would lead to insufficient discussion, while a larger sample can be chaotic, difficult to manage by the moderator and the participants may feel they did not get sufficient opportunities to speak.

### Instrument

The questions of the interview were adapted from a validated questionnaire.^16^ The students from both focus groups were asked semi-structured questions. The interviews were conducted by the principal investigator (AS) and the two secondary investigators (MHBS and SP). The questions focused on 12 themes: learning objectives, syllabus coverage, practical skill assessment, deficiencies, confidence, competence, fairness, learning, time restrains, organisation of exam, comfort and stress. Table 2. Shows the frequencies of most repeated terms and their organisation into various themes.

### Procedure

The studies with the focus groups were carried out in a teaching room that was familiar to the students. The facilitator gave a brief introduction on what the discussion session was about and informed the students about the confidentiality of the report. Thereafter, the facilitator introduced the participants to various dimensions/topics of the discussion interview one by one in the form of questions. The facilitator let the discussion proceed till a saturation of themes or perceptions was reached. Each session lasted for ~45 min. The discussions/interviews were recorded using audio recorders. The recordings were transcribed verbatim into a word processing format by the primary and secondary investigators.

A general inductive approach was used to analyse the data in two stages. First, an open coding was done whereby the transcriptions were read from line to line to identify emerging themes and an initial coding framework was made (AS). Second, the categories were further reduced in number by grouping them together by the secondary investigator/supervisor (MHBS). The research data was then organised according to the finalised themes. The supervisor then reviewed: the focus group transcript, analysed data and emerging themes.

The educational background and perspectives of the primary investigator (AS) and a secondary investigator (SP) and effect of these issues are worth noting in relation to this study as they were residents/house officers at the colleges’ attached dental hospital at the time of the study. The influence of the researcher’s own experiences and perception may be seen both as a strength and limitation of the study.^25^ As an insider to the context, AS and SP were able to gain access and establish rapport with the participants: they had a good understanding of the participants’ learning environment and the participants seemed to be willing to share their experiences honestly compared with an outsider. However, to analyse the data objectively, a level of disconnection was also necessary. To avoid making analytical assumptions based on close acquaintance with participants, proximity to research context and personal preconceptions, following steps were taken. First, a focus on participants’ view was maintained by emphasising on student voices in research reporting: the research findings were considered and analysed in relation to existing literature: and, although, AS conducted the initial thematic coding, this was checked by the supervisor, who had a diverse educational, epidemiological and dental public health expertise.

**Figure 1:**
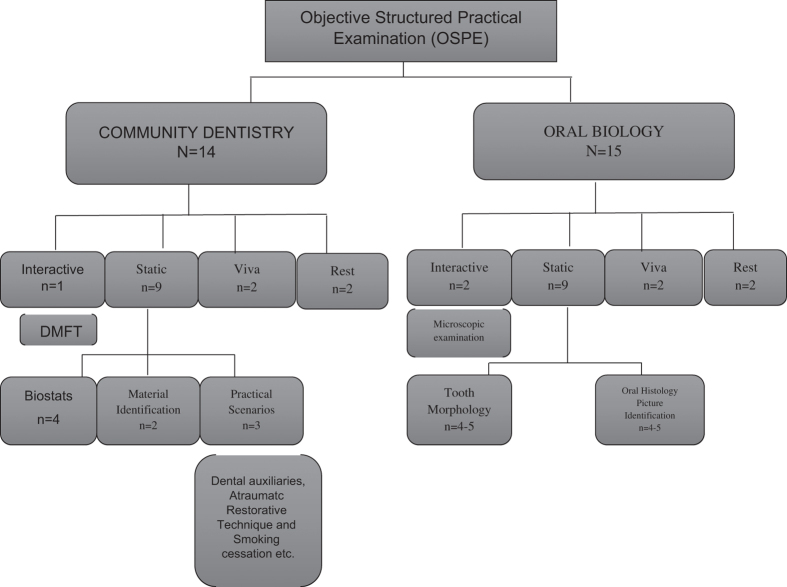
Format for OSPE in Islamic International Dental College, where *N* is the total number of stations and *n* is the number of stations in each category.

**Figure 2:**
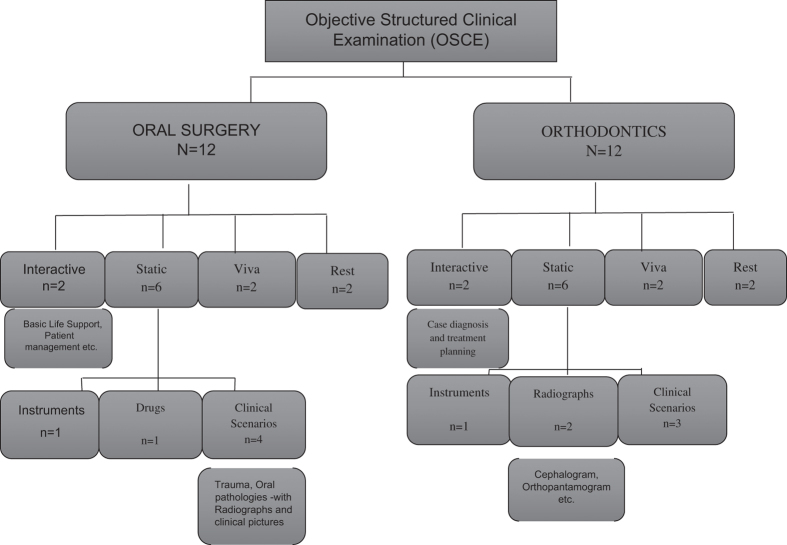
Format of OSCE in Islamic International Dental College, where *N* is the total number of stations and *n* is the number of stations in each category.

The format of OSPE and OSCE in our institution for these subjects is outlined in Figures 1 and 2.

## Results

A total of 16 participants were interviewed. The participants were divided into two focus groups (OSPE and OSCE), each including eight individuals.

The participants shared their views regarding strengths and the limitations of the OSPE and OSCE under eight different topics. They also gave their suggestions to improve the assessment strategies. The main themes related to the design, content, organisation and weakness of the OSPE and OSCE are discussed in this paper.

### OSPE Focus group

#### Curriculum

The participants generally agreed with the point that the OSPE comprehensively covers the learning objectives for both CD and OB. The students reported that the OSPE tests the underlying core concepts in a more concise manner than simply examining theoretical knowledge.

*‘If you ask me the definition of health, I wouldn’t know but when I gave it on OSPE I simply remembered this …. There was more concept than definition’*

(DS 1)

On the other hand, some of the students did opine that since, the time available at individual stations is relatively short; it does not cover the depth of the curriculum comprehensively.

*‘Syllabus was very vast, putting that into a one-hour exam is easier, putting that into 2–3 min stations, there is only so much you can do’*

(DS 4)

The students also reported that the main reason that the OSPE for OB and CD was not comprehensive enough because it relied a greater focus on certain areas of the curricular content.

*‘…it focused more on tooth morphology’-oral biology*

(DS 3)

*‘…there was more emphasis on the 3rd quadrant (research methodology)’-community dentistry*

(DS 7)

#### Skills assessment

The student reported that the OSPE for CD did include assessment of the students’ psychomotor practical skills. However, there were important practical skills that were not tested.

*‘In community we had to do DMFT which is practical but more practical skills could have been tested like probing and demonstration of brushing techniques’*

(DS 2)

The OSPE tests the concepts of the students and any confusion they may have regarding the subject becomes evident

*‘In first OSPE of community we were not ready because we didn’t have the concepts’*

(DS 8)

While some students believed that OSPE was similar to the theory exam for both subjects, others opinioned that this was the case only for CD. One student stated:

*‘…we had to calculate the odds ratio in both the theory and OSPE; the only difference is that OSPE is a time based exam’*

(DS 1)

Other students disagreed with the above point stating that as compared to the theoretical exam (comprised of short essay questions and multiple choice questions); the OSPE is more concept-oriented.

Regarding their assessment of their competencies, the students stated:

*‘..You are asked on the spot to apply a test, so your competencies are assessed’ community*

(DS 6)

*‘..We are just given a few instruments to identify so our competencies are not checked’ oral biology *

(DS 4)

The students were of the opinion that they should be taught more realistically rather than giving them ideal scenarios and diagrams so their competencies can be assessed in a better way.

#### Confidence building

The students reported that OSPE improved the confidence of the students by requiring them to work under pressure due to timed stations. However, they also reported that traditional vivas, although biased, are better at confidence building, in comparison to OSPE.

*‘..You learn to answer in a short time……. vivas are better for developing confidence because we are facing the examiners directly………. vivas are biased, they are unfair and there is only so much you can say in 7 min’*

(DS 3)

#### Fair assessment tool

The students generally agree with the point that the OSPE is a fair method of assessment as everybody is required to answer the same questions in a fixed amount of time. However, the students also felt that the OSPE was not fair for such students who were slow learners and had problems performing under high pressure circumstances. A few suggestions were put forth to solve this issue. First, extra time may be given to such students. A better suggestion was that such students should be identified before the summative assessment and counselling should be done to alleviate their stress issues. The stress management problem of these students is exacerbated by the high pressure, timed stations of the OSPE. Prior feedback and mentoring should be done for all such students so that they are able to handle pressure situations more efficiently.

One student reported:

*‘…I’ve seen many students fail just for this matter that they were unable to finish the test on time’*

(DS 2)

#### Impact on learning

OSPE was reported to have changed the way students study. The students said that they challenged themselves while studying for the OSPE.

One student reported:

*‘In OSPE we studied in a whole different way…we studied together and observed a shift in our style….. We gave each other scenarios and whoever made the toughest one, he gets away. So we challenged ourselves, we thought out of the box’*

(DS 5)

The students believed that the OSPE stimulated and modified their learning and is less frustrating, more appealing and easier to learn.

Another student reported:

*‘We study according to the way we are going to be assessed; we focus more on the diagrams and pictures now…………………. Compared to conventional exams, learning is better with OSPE and is more concepts-based’*

(DS 6)

#### Stress

The general perception of the students was that the OSPE is less stressful than the traditional vivas. The male students expressed greater concerns with being stressed during the OSPE, in comparison to the female students in the focus group. The reasons described for stress were less time at each station—the general perception being that the OB tasks at each station were not timed adequately and required more time than the allocated time.

*‘My first question took a lot of time and I was lagging behind, so it took some time for me to get back on track. It really brought down my confidence’*

(DS 1)

#### Organisation of exam

The OSPEs were well organised. Students did point out that there should be extra sheets available for rough work required at some of the stations

*‘There should’ve been extra sheets for rough work because I made my paper untidy’*

(DS 7)

#### Examiners calibration

The examiners training and calibration was raised as a point of concern.

One student reported:

*‘I did hear the examiner screaming at some students, that is talking in a very loud tone, basically she was scolding the students, so we all got distracted’*

(DS 8)

With reference to the viva station, the students also though that the questions asked by the OB examiners varies between students. The viva station for OB was placed in the middle of the hall where the OSPE was being conducted and there was no partitioning, such that the viva discussions could be heard by other students making the whole assessment process less transparent.

### OSCE Focus group

#### Curriculum

The FGs agreed that the OSCE comprehensively covered most of the learning outcomes for both Orthodontics and Oral Surgery.

#### Skills assessment

The FGs strongly expressed their opinion regarding lack of clinical and practical skills being tested during the OSCE. According to a FG:

*‘…….. in orthodontics OSCE skills are not judged as patients’ profile pictures were shown and they demanded the treatment plan, so in this way more theoretical knowledge is applied rather than clinical skills,’*

Another FG commented on the Oral Surgery OSCE:

*‘… no extractions were performed, no practical skills observed however only patient dealing was tested’* (FG 3)

Regarding competencies being assessed the FGs opined:

*‘…competency is related to clinical experience and clinical work; it does not come with theoretical knowledge alone. It is based on skills that you have acquired’*

(FG 2)

*‘…Competency recognizes the ability of the student to be good and competent in a certain area, the OSCE does not assess it, it can only tell how good you are at dealing with the patient, that is not about how good are you at your clinical work’*

(FG 8)

#### Confidence

The FGs agreed that the OSCE was a *‘better and good way to develop confidence’* (FG 6).

#### Fair assessment tool

The OSCE was believed to be a fair assessment tool.

*‘It is same and standardised for the entire class as compared to conventional vivas in which some students had long while others had short vivas; therefore OSCE exam is much fairer than conventional vivas’*

(FG 4)

#### Impact on learning

The FGs thought that the OSCE had a greater impact on students’ learning, in comparison to traditional practical exam. The explanation given was that in contrast to the traditional practical exam, the OSCE covered a greater range of competencies and topics.

*‘.. Even if we cover the entire syllabus before viva we would not be confident enough whether we would be able to perform OSCE well or not’*

(FG 1)

Regarding future considerations, the graduates were very hopeful that they get a good practice as they have to follow a protocol to help them understand how to treat patients in routine practice of dental clinics.

*‘In a way you have to thoroughly prepare yourself, therefore you have to study extensively and frequently practice as well’*

(FG 7)

#### Stress

Perceptions regarding confidence levels varied among the FGs. Some reported being confident while others were stressed out during the OSCE. The most important stressor that was pointed out was lack of time at each station.

One graduate stated:

*‘You couldn’t properly explain your point to examiner and buzzer would already go off’* (FG 3)

*‘Every student should be allowed to say next, when they don’t know the answer’*

(FG 6)

They also stated that OSCE and viva stations should be separate; there should be no lengthy discussions, and no cross questioning.

#### Organisation of exam

The OSCE was reported to be well organised for both exams, except for lack of time at each station.

A FG commented:

*‘There was less time for a few stations like for writing the mechanism of action of drugs’* (FG 5)

#### Examiners calibration

The examiners were thought to be a little strict with respect to their attitudes. Also, the students disapproved of examiners using cell phones while conducting vivas, as it gave a very non-professional and a non-serious outlook.

*‘They should be supportive, have the ability to extract the answer form the student, should not be bias and should ask same questions from each and every student.’*

(FG 1)

## Discussion

This study explores the dental students’ perceptions regarding OSPE and OSCE. An advantage of the OSCE is the flexibility and versatility made possible by the multiple station design enabling a range of competencies to be examined.^26^ The students in the present study reported that the OSPE did not associate an appropriate weightage to all the topics of the curriculum by laying a greater emphasis on selected topics. However, Al-Mously *et al.* reported contrary opinion by their students.^16^ OSPEs should be designed such that they cover all topics adequately and assess the students’ practical skills, problem solving, numeracy, communication skills and other graduate attributes without large amount of paper work.^27^

OSPE/OSCE has been accepted as a reliable and valid method of skills assessment.^16,27,28^ It promotes critical thinking among students enabling them to work on the competencies for which they would be assessed upon. This critical analysis, in turn, results in students weighing their strengths and weaknesses.^27^ However, our study reported that students were not completely satisfied with the way their practical skills were assessed through OSCE. This may be explained by the lack of critical application scenarios at the different OSCE stations.

According to the FGs, undergraduate orthodontics curriculum being theory based did not require assessment of clinical skills in OSCE. In contrast, Oral Surgery OSCE underscores the assessment of skills such as local anaesthesia administration, behavioural management and extractions, however they were not evaluated. Although FGs did explain that these skills were tested in their clinical rotations during the academic year. This simply translates into the fact that it is important how the OSCE stations are designed. The OSCE purports to assess psychomotor skills of the students. If the stations do not have any design to assess clinical skills, the purpose of conducting the OSCE may not be fully achieved. Such kind of OSCE is not a good tool to assess skills as it lacks the clinical authenticity and subsequently, may mislead to judge the competencies of the student.^29^

Students’ feedback showed that although OSPE teaches them to work under pressure, it does not help develop confidence better than a traditional viva. However some studies report a contrary finding.^16^

Overall OSPE is viewed as a fair assessment tool.^16,28,30^ However, the students perceived an issue that OSPE would not be fair for students who are slow learners and take time to understand the task. Similar concerns were raised by Derek *et al.* who pointed out that some nervous students need reassurance if previously reliant on written work.^27^ Frantz *et al.* reported that the students in his study felt they should be steered in the right direction if they misinterpret the question.^12^ The FGs deemed OSCE to be fair.

Assessment drives learning. The students in the present study thought they saw a shift in their style of learning; they started challenging themselves and thinking out of the box. They believed learning for the OSPE and OSCE are better than that for the conventional exams. Regarding future considerations, the graduates were very optimistic as OSCE had an impact on future learning as it differs from conventional vivas that pose great deficiencies in the learning of professional skills.

OSCE was perceived as a stressful exam by FGs. Time limitation was reported as a major drawback as supported by previous literature.^12,16,28,30^ Less amount of time to complete a station increases stress among students. The examiners should consult each other to balance the time given at each station according to the tasks at hand. Our findings show that OSPE is less stressful than the traditional exam which is contrary to a study conducted in India in which the students felt more pressure in OSPE than the traditional exam.^31^ Literature shows that OSPE does create pressure initially but as the assessment begins the anxiety tends to decrease^32^ and the students generally perform well.^33^

Planning and organisation is a key to success.^27^ One problem with many dental colleges is that teachers often spend more time on preparing lessons and teaching them, than they do on assessing competencies. Time spent on improving the assessment would be richly repaid with improved students; learning.^20^

Examiners’ calibration was one of the major issues raised in this study. The examiners were not consistent with their questions and had attitudes that greatly affected students’ performance. Frantz *et al.* raised similar concerns in which the staff felt that there was a need for consistency from one student to the next.^12^ The examiners should be briefed beforehand on their role in an OSPE/OSCE examination to maintain the reliability of the tool and prevent subjectivity.

## Conclusion

OSPE and OSCE have evolved to become a much more valid and reliable assessment strategy, in comparison to the conventional viva and long case examinations. The manner in which OSPE and OSCE are conducted ensures the assessment of many dental competencies. However, several essential competencies were not adequately assessed in the setting of the present study. Although, OSPE and OSCE are much better ways of assessing the competencies of dental students than the conventional examinations, meticulous care must be given to the designing of the respective OSCE and OSPE to avoid multiple biases as have been reported in this study.

## Figures and Tables

**Table 1:** Skills tested on an OSPE or OSCE and relevant modes of assessment (adapted and modified from Prozesky

*Skill/enabling factor to be examined*	*Description*	*Suitable assessment method*
Manual skill	Performing a particular procedure, e.g., extraction or suture placement.	Student has to perform the procedure (extraction or suturing) on a patient while the examiner watches and marks his/her performance with a checklist.
Communication skill	Educating the family on a particular matter, e.g., prevention of oral cancer.	Student has to educate the family regarding prevention of oral cancer while the examiner watches and score according to a checklist.
Decision making skill	Diagnosing and treating a case, e.g., trauma.	The student is presented with a patient suffering from trauma. She/he has to examine the patient and make a diagnosis, while the teacher watches. The teacher can also give the students a written case study, which gives the history and examination findings, and ask them how they would manage the patient.
Knowledge	Knowledge of signs, symptoms, anatomy, spread, medication, prevention and so on.	Written examination with short questions, MCQs, essay questions. Oral examination.
Attitude	An attitude of concern and caring.	The teacher observes the students as she/he works in the clinic. After a week or so the teacher uses a checklist to make a final assessment of the students’ attitude.^20^

**Table 2:** Frequency of repeated terms in OSPE and OSCE and their organisation into various themes

*No.*	*Themes*	*OSPE*	*OSCE*	*Frequency*	*Total*
1.	*Curriculum*				
	Syllabus	+	++	3	7
	Repetition	+++	+	4	
					
2.	*Skills assessment*				
	Practical/clinical skills	++	+++	5	12
	Concepts	+++	+	4	
	Competence	++	+	3	
					
3.	*Confidence*				
	Confidence	++	+	3	6
	Comfort	++	+	3	
					
4.	*Fairness of exam*	++	++	4	4
					
5.	*Impact on learning*				
	Improved learning	+	+	2	5
	Future learning	+	++	3	
					
6.	*Stress*				
	Stress	++++	++++	8	17
	Time	++++++	+++	9	
					
7.	Organisation of exam	++	++	4	4
					
8.	Examiners’ calibration	+++	++++++++	8	11
